# Supplementary material for: The Alleviation of Dextran Sulfate Sodium (DSS)-Induced Colitis Correlate with the logP Values of Food-Derived Electrophilic Compounds
Source: Antioxidants (Basel). 2022 Dec 5;11(12):2406. doi: 10.3390/antiox11122406 (PMC9774124; doi:10.3390/antiox11122406)
Supplement: Supplementary file 1 [file antioxidants-11-02406-s001.zip › antioxidants-2055049-supplementary.pdf]

Supporting information

Table S1 Analysis of the interaction between FECs and BTB domain of Keap1.

Table S2 The data of correlation analysis between molecular characteristic values of FECs and DAI scores in colitis.

Table S3 The data of correlation analysis between molecular characteristic values of FECs and pro-inflammatory cytokines in colon.

Table S4 Correlation analysis between molecular characteristic values of FECs and oxidative indicators in colon.

Table S5 The data of correlation analysis between molecular characteristic values of FECs and the protein expression levels of Nrf2, NQO1 and HO-1 in colon.

Figure S1 Correlation analysis between molecular characteristic values of FECs and DAI scores in colitis.

Figure S2 Correlation analysis between molecular characteristic values of FECs and pro-inflammatory cytokines in colon.

Figure S3 Correlation analysis between molecular characteristic values of FECs and oxidative indicators in colon.

Figure S4 Correlation analysis between molecular characteristic values of FECs and the protein expression levels of Nrf2, NQO1 and HO-1 in colon.

Table S1 Analysis of the interaction between FECs and BTB domain of Keap1.

Compound name	amino acid	Interaction force
**FMA**	Tyr85	Hydrogen bond
	His129	Hydrogen bond
	His154	Hydrogen bond
	Asn157	Hydrogen bond
	Gln86	Van der Waals
	Gly127	Van der Waals
	Ile128	Van der Waals
	Val132	Van der Waals
	Val155	Van der Waals
	Gly158	Van der Waals
**ISO**	Lys131	Hydrogen bond
	Arg135	Hydrogen bond
	Cys151	Hydrogen bond, Pi-alkyl
	Tyr85	Van der Waals
	Val132	Van der Waals
	Gly148	Van der Waals
	His129	Pi-cation
	His154	Pi-Pi stacked
**CA**	Tyr85	Hydrogen bond
	His154	Hydrogen bond, Pi-Pi stacked
	Val132	Van der Waals
	His129	Pi-cation
	Lys131	Pi-alkyl
	Cys151	Pi-alkyl

**FA**	Glu149	Hydrogen bond
	Lys150	Van der Waals
	Val152	Van der Waals
	His154	Van der Waals
	Leu175	Van der Waals
	Leu153	Pi-alkyl
**SFN**	His129	Hydrogen bond
	Tyr85	Van der Waals
	Gly127	Van der Waals
	Ile128	Van der Waals
	Val132	Van der Waals
	Leu153	Van der Waals
	Asn157	Van der Waals
	Gly158	Van der Waals
	His154	Pi-sulfur
**CGA**	Lys131	Hydrogen bond
	Arg135	Hydrogen bond
	Gly148	Hydrogen bond
	Tyr85	Van der Waals
	Val132	Van der Waals
	Met147	Van der Waals
	Lys150	Van der Waals
	His129	Pi-cation
	His154	Pi-Pi stacked
	Cys151	Pi-alkyl

Table S2 The data of correlation analysis between molecular characteristic values of FECs and DAI scores in colitis.

	DAI score	log*P*	Keap1 affinity	Electrophilic index
DAI score	1	-0.738*	-0.592	0.392
log*P*	-0.738*	1	-0.12	0.278
Keap1 affinity	-0.592	-0.12	1	-0.754*
Electrophilic index	0.392	0.278	-0.754*	1

*** P < 0.05**

Table S3 The data of correlation analysis between molecular characteristic values of FECs and pro-inflammatory cytokines in colitis.

	log*P*	Keap1 affinity	Electrophilic index	TNF-α	IL-1β	IL-6
log*P*	1	-0.012	0.278	-0.544	-0.618	-0.577
Keap1 affinity	-0.012	1	-0.754*	-0.45	-0.181	-0.803*
Electrophilic index	0.278	-0.754*	1	0.561	0.179	0.442
TNF-α	-0.544	-0.45	0.561	1	0.785*	0.646
IL-1β	-0.618	-0.181	0.179	0.785*	1	0.429
IL-6	-0.577	-0.803*	0.442	0.646	0.439	1

*** P < 0.05**

Table S4 Correlation analysis between molecular characteristic values of FECs and oxidative indicators in colon.

	logP	Keap1 affinity	Electrophilic index	T-AOC	MPO	GSH-px	SOD	MDA
logP	1	-0.012	0.278	0.607	-0.371	0.621	0.517	-0.402
Keap1 affinity	-0.012	1	-0.754*	0.693	-0.631	0.59	0.509	-0.221
Electrophilic index	0.278	-0.754*	1	-0.385	0.747	-0.31	-0.476	0.377
T-AOC	0.607	0.693	-0.385	1	-0.797*	0.711*	0.486	-0.588
MPO	-0.371	-0.631	0.747*	-0.797*	1	0.565	-0.611	0.714
GSH-px	0.621	0.59	-0.31	0.711	-0.565	1	0.817*	-0.563
SOD	0.517	0.509	-0.476	0.486	-0.611	0.817*	1	-0.332
MDA	-0.402	-0.221	0.377	-0.588	0.714	-0.563	-0.332	1

*** $P < 0.05$**

Table S5 The data of correlation analysis between molecular characteristic values of FECs and the protein expression levels of Nrf2, NQO1 and HO-1 in colitis.

	logP	Keap1 affinity	Electrophilic index	Nrf2	NQO1	HO-1
logP	1	-0.012	0.278	0.922**	0.737*	0.759*
Keap1 affinity	-0.012	1	-0.754*	0.173	0.468	0.266
Electrophilic index	0.278	-0.754*	1	-0.069	-0.382	-0.18

Nrf2	0.922**	0.173	-0.069	1	0.888**	0.842*
NQO1	0.737*	0.468	-0.382	0.888**	1	0.953**
HO-1	0.759*	0.266	-0.18	0.842*	0.953**	1

* P < 0.05, ** P < 0.01

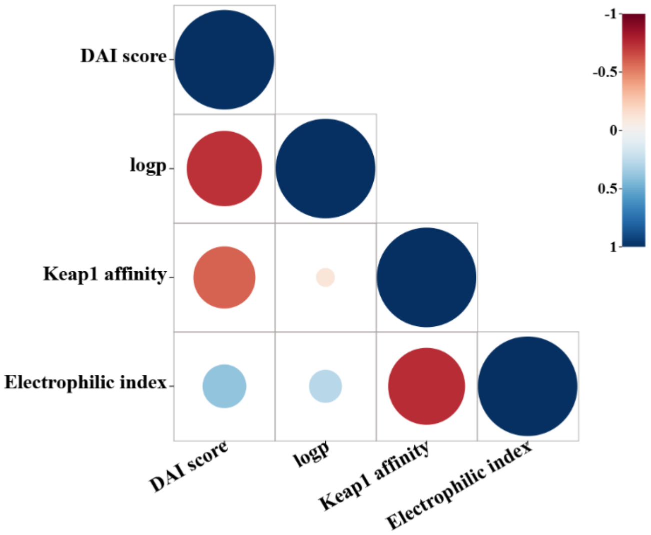

Figure S1 Correlation analysis between molecular characteristic values of FECs and DAI scores in colitis.

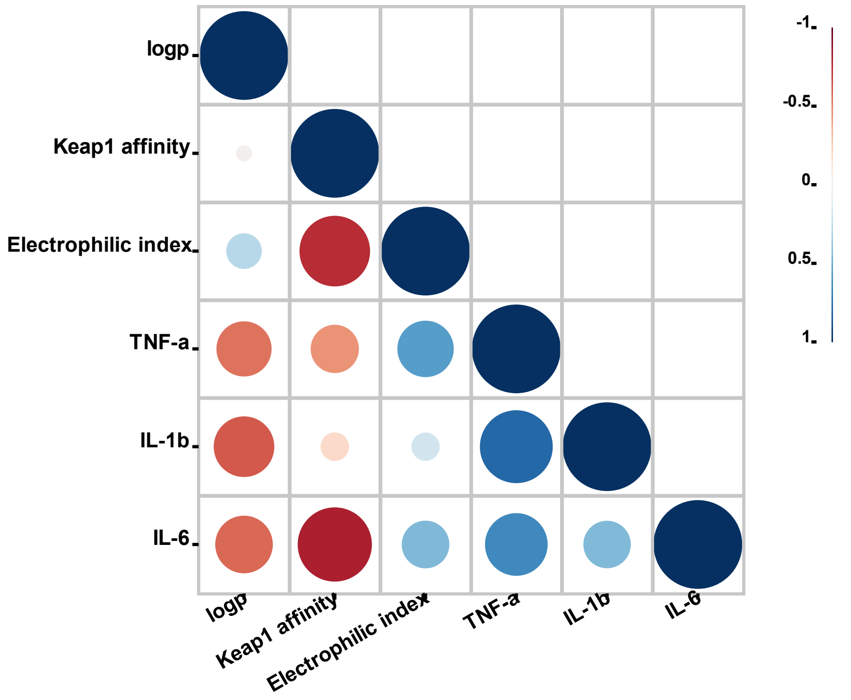

Figure S2 Correlation analysis between molecular characteristic values of FECs and pro-inflammatory cytokines scores in colitis.

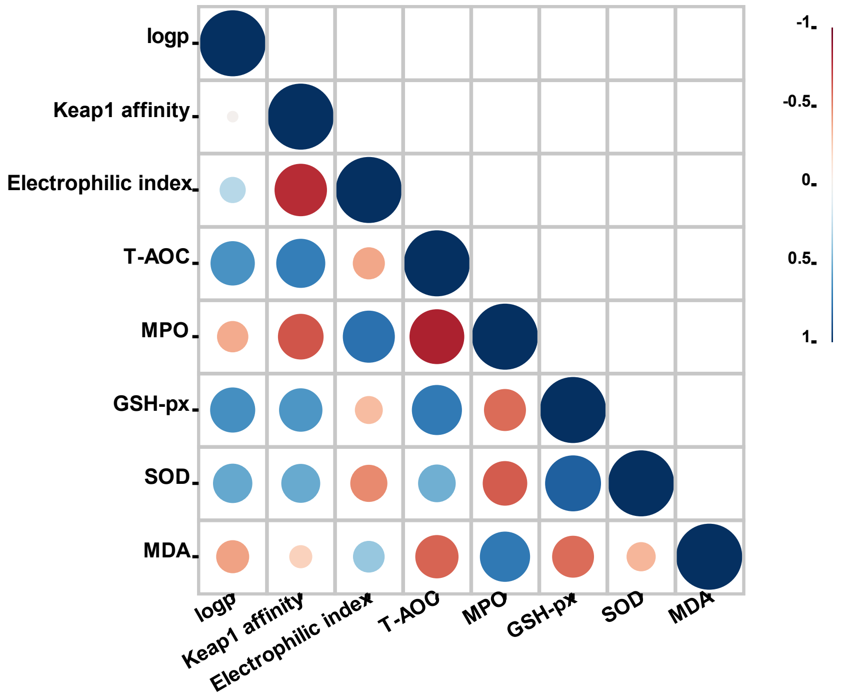

Figure S3 Correlation analysis between molecular characteristic values of FECs and oxidative indicators in colon.

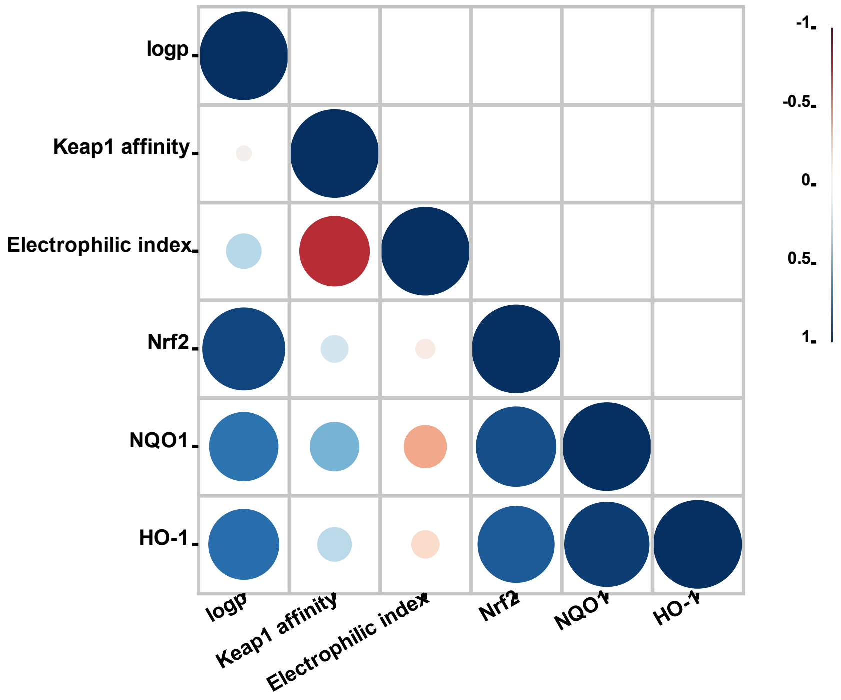

Figure S4 Correlation analysis between molecular characteristic values of FECs and the protein expression levels of Nrf2, NQO1 and HO-1 in colon.
